# Update on the management of Barrett’s esophagus in Austria

**DOI:** 10.1007/s10353-017-0504-y

**Published:** 2017-12-04

**Authors:** M. Riegler, I. Kristo, M. Nikolic, E. Rieder, S. F. Schoppmann

**Affiliations:** 1 Reflux Medical Vienna, Vienna, Austria; 2 Department of Surgery, Upper-GI-Service, Comprehensive Cancer Center, GET-Unit, Vienna General Hospital, Medical University of Vienna, Vienna, Austria

**Keywords:** Barrett’s esophagus, Esophageal adenocarcinoma, Antireflux surgery, Esophageal manometry, Squamo-oxyntic gap, Cardiac mucosa, Low-carbohydrate diet

## Abstract

**Background:**

Barrett’s esophagus (BE) is the premalignant manifestation of gastroesophageal reflux disease (GERD). Radiofrequency ablation (RFA) with and without endoscopic resection (ER) is a novel treatment for BE.

**Methods:**

Here we present a single-center update of the recommendations of a recent (June 2015) interdisciplinary expert panel meeting on the management of BE with dysplasia as well as cancer-positive and cancer-negative BE. We conducted a PubMed search of studies published in 2016 and 2017 on the topic of BE and RFA.

**Results:**

Our update reconfirms that BE positive for T1a cancer as well as low- and high-grade dysplasia justifies the use of RFA ± ER, offering an 80–100% rate of BE clearance. RFA ± ER of dysplastic BE is tenfold more effective for cancer prevention when compared with surveillance. Risk factors for recurrence and follow-up treatments include baseline histopathology (dysplasia/T1a cancer), esophagitis, hiatal hernia >3 cm, smoking habits, BE segments >3 cm, and >10 years of GERD symptoms. A baseline diagnosis for dysplasia and T1a cancer should include a second expert pathologist opinion. Recent data justify the use of RFA for nondysplastic BE only in controlled clinical trials. Antireflux surgery can be offered to those with function-test-proven, GERD-symptom-positive BE before, during, or after RFA ± ER. Additionally, there is growing evidence that the intake of a sugar-rich diet is positively correlated with the development of GERD, BE, and cancer.

**Conclusion:**

RFA ± ER should be offered for dysplastic BE and T1a cancer after ER as well as for nondysplastic BE with additional risk factors in controlled trials. Antireflux surgery can be offered to patients with function-test-proven GERD-symptom-positive BE. Diet considerations should be included in the management of GERD and BE.

## Introduction

**Fig. 1:**
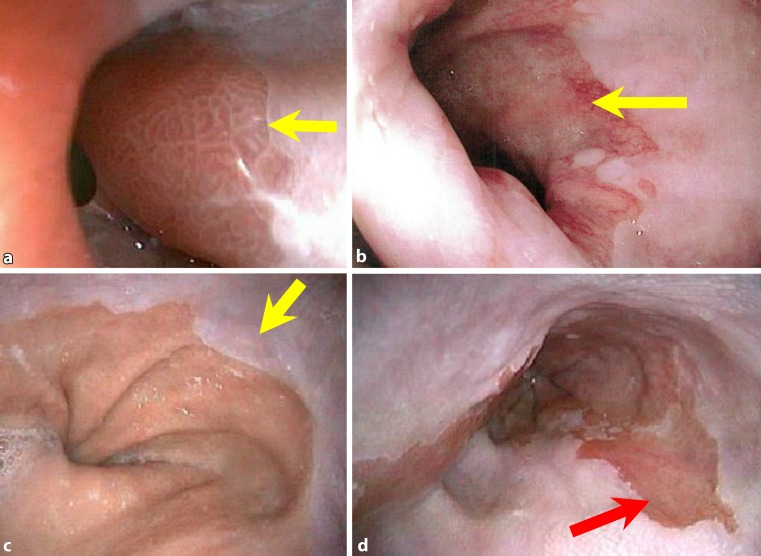
Antegrade endoscopic images (**a**–**d**) of columnar lined esophagus distal to the endoscopic squamocolumnar junction (SCJ; *arrows*). Note the presence of typical geometric pattern of the columnar lining distal to the SCJ in **a**, typical for cardiac-type mucosa. Biopsies obtained from the SCJ in cases **a**–**d** were positive for Barrett’s esophagus without dysplasia. Images obtained using Storz technology

**Fig. 2:**
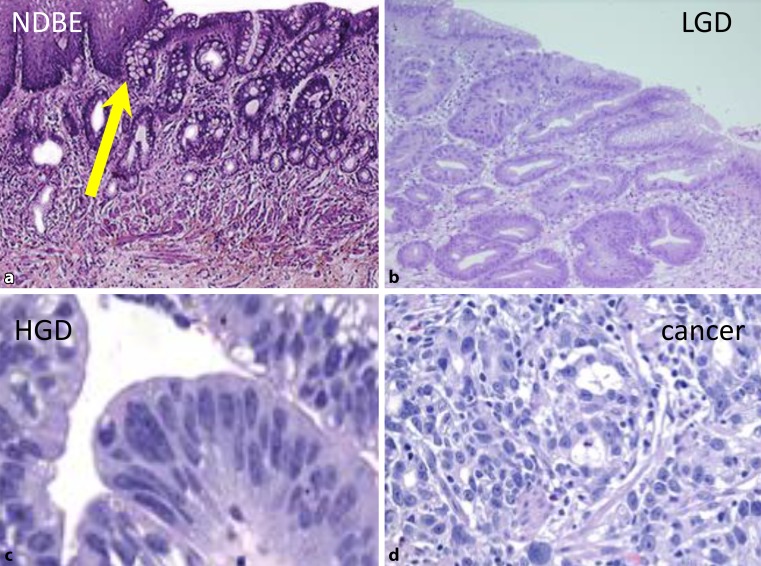
Histopathology of nondysplastic Barrett’s esophagus (**a**, *NDBE*), low-grade dysplasia (**b**, *LGD*), high-grade dysplasia (**c**, *HGD*), and (**d**) cancer. *Yellow arrow* in **a** indicates goblet-cell-positive mucosa adjacent to the squamocolumnar junction. Goblet cells are the hallmark for the diagnosis of Barrett’s esophagus without dysplasia. H&E stain, × 50 in *NDBE*, *LGD*, *cancer;* × 100 in *HGD.* (Courtesy of Prof. Fritz Wrba, Vienna)

Barrett’s esophagus (BE) represents the morphologic premalignant manifestation of gastroesophageal reflux disease (GERD), which develops as a consequence of the dysfunction and failure of the antireflux mechanism within the lower end of the esophagus ([1]; Fig. 1). Via low- (LGD) and high-grade dysplasia (HGD), nondysplastic BE may progress toward adenocarcinoma of the esophagus (risk approx. 0.5% per year; [2, 3]; Fig. 2).

**Fig. 3:**
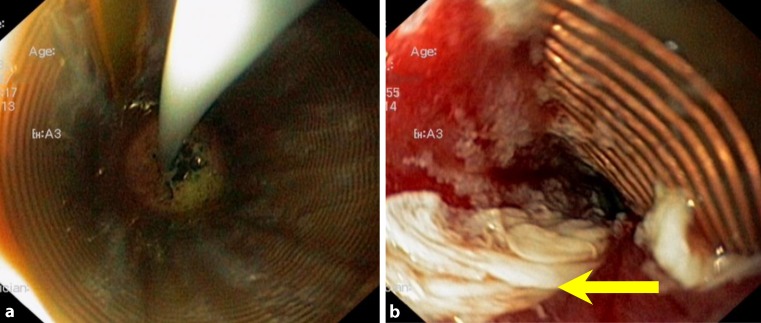
Antegrade endoscopic view during RFA in the distal esophagus, using the catheter-mounted balloon (RFA 360). **a** View through the treatment balloon during the delivery of the radiofrequency energy to the tissue, as described in the text. **b** Image after RFA treatment with the balloon deflated. The *yellow arrow* indicates the ablated mucosal tissue. Note the red surface of the submucosa indicating adequate ablation

Radiofrequency ablation (RFA; Fig. 3) and endoscopic mucosal and submucosal resection are treatment modalities for the durable eradication of BE, dysplasia, and early cancer and have been demonstrated to foster cancer prevention [2, 3].

A recent Austrian multidisciplinary expert panel meeting held in Vienna in June 2015 summarized recommendations for the management of BE [3]. Here we aim to summarize and update the recommendation by inclusion of relevant studies published in 2016 until December 2017 from the viewpoint of a high-volume center.

## Methods

Based on the recommendations of the recent expert panel meeting we conducted a search of PubMed and Scopus including the following keywords: Barrett’s esophagus, endoscopy, endoscopic mucosal and submucosal resection, anti-reflux surgery, fundoplication, gastroesophageal reflux disease, histopathology, LINX lower esophageal sphincter augmentation system, radiofrequency ablation, and surveillance. The analysis included meta-analyses, review articles, and retrospective follow-up studies. Statistical analyses were not applied.

## Results

Our search aimed to update the current data on to the diagnosis, therapy, and follow-up of patients with GERD and BE.

### Diagnosis of BE

The diagnosis of BE is established by histopathology of endoscopic biopsies obtained from the esophagus and esophagogastric junction (EGJ), using the novel Chandrasoma classification [1]: The normal lining of the esophagus and the proximal stomach are stratified squamous and oxyntic mucosa, respectively [1]. Owing to the failure of the antireflux mechanism within the lower end of the esophagus, reflux occurs and stimulates the metaplasia of the squamous lined mucosa [1, 2]. Thereafter, the columnar lined esophagus (CLE) develops and is interposed between the squamous lining of the esophagus and the oxyntic mucosa of the proximal stomach, i. e., this condition is termed the squamo-oxyntic gap (SOG) and represents the morphologic proof of GERD [1].

According to the Chandrasoma classification, CLE includes cardiac mucosa (CM: mucus-cell-only epithelium), oxyntocardiac mucosa (OCM: mixture of mucus and parietal cells within the subfoveolar region of the glands), nondysplastic BE (cardiac mucosa with goblet cells; intestinal metaplasia: IM), LGD, HGD, and cancer ([1, 3]; Fig. 2). Over time, further dysfunction of the antireflux mechanism aggravates the reflux and increases the length of the CLE, i. e., the length of the SOG. BE affects 20–30% of individuals with symptoms of GERD [2]. The diagnosis of LGD, HGD, and early cancer should be confirmed by an expert pathologist (second opinion; [4, 5]).

### Endoscopic BE treatment

Endoscopic therapies for the elimination of BE include endoscopic mucosal and submucosal resection for the removal of nodules and tumors within the CLE [3]. RFA represents a modern endoscopic therapy for the removal of endoscopically flat visible CLE containing BE, LGD, HGD, and early cancer ([2, 3], Figs. 2 and 3). The radiofrequency energy is applied to the CLE under endoscopic vision from self-sized balloon catheter-mounted electrodes (RFA 360; Fig. 3), an endoscope tip (RFA 90, 60), or working channel-mounted electrodes (“eagle” device; [2, 3]).

### Management of dysplastic BE

The interdisciplinary expert panel meeting recommended RFA ± ER for T1a cancer, HGD, and LGD [3]. An accurate baseline diagnosis is of profound importance for the disease management [3]. Recent studies have confirmed these recommendations.

Duits et al. [4] retrospectively examined the effect of RFA for elimination of BE with LGD in 255 patients after 42 months (range: 25–61; 3–5 years of follow-up; SURF trial data). During the follow-up, 18% of the patients (45/255) progressed to esophageal cancer.

The study showed the vital importance of accurate baseline histopathology for the assessment of risk for disease progression after the RFA therapy. As such, the odds for progression increased 8–13-fold, and 22–38-fold, when baseline LGD (prior to RFA) was assessed by each of the pathologists or reconfirmed by all of them, respectively [4].

Guthikonda et al. [6] conducted a retrospective analysis of 306 patients who underwent RFA for the elimination of dysplastic BE between March 2006 and June 2015. At the first follow-up endoscopy (<1 year) after RFA elimination, BE and dysplasia were assessed in 85 and 88.9% of cases, respectively. In all, 218 patients continued with follow-up to assess disease recurrence. During a mean time to recurrence of 1.88 years, 24% (*n* = 52) of the patients developed recurrence of IM, this translates to an incidence of 9.6% per year for IM after RFA. Recurrences were assessed in biopsy samples obtained from the esophagus, cardia, and both in 63%, 33%, and 4% of cases, respectively. Following repeated RFA, 58% (*n* = 30) of those with recurrence were free from IM. Those positive for IM were enrolled in additional follow-up RFAs. During the study, four patients developed cancer. Risk factors for progression and development of cancer included baseline HGD and longer CLE segments.

Cameron et al. [7] retrospectively examined the recurrence rate following one to six RFA ± endoscopic mucosa resection (EMR) sessions in 137 individuals for the treatment of BE low- (25%), high-grade (54%), and intramucosal adenocarcinoma (21%; AIM dysplasia trial). Elimination of dysplasia and intestinal metaplasia was achieved in 88%, 92%, 100%, and in 69%, 74%, and 81% of patients after 1, 2, and 3 years, respectively. Thus, Kaplan–Meier estimates were 58%, 88%, and 95% and 41%, 72%, and 82% for dysplastic and nondysplastic BE after 1, 2, and 3 years, respectively. Dysplasia (HGD or LGD) was the most advanced stage of recurrence, and none of the patients progressed to or developed cancer. In all, 80% of the recurrences were assessed in biopsy samples obtained from the EGJ [7].

A recent meta-analysis examined the efficacy of RFA ±EMR for the management of dysplastic and nondysplastic BE.

Luigiano et al. [8] confirmed that 1–3 years after RFA dysplastic and nondysplastic BE were eliminated in 60–100% and 60–90% of the cases, respectively. Furthermore, RFA was demonstrated to be superior to surveillance for the management of LGD. The cumulative 3‑year risk of LGD to progress to HGD and cancer was 33% and 2.9% in the RFA ± EMR treatment group vs. surveillance, respectively [8].

Following RFA ± EMR, the reported elimination rates for HGD and IM were 70–100% and 60–96%, respectively [8].

In their meta-analysis, Fujii-Lau et al. [9] included 39 out of 3311 studies and examined the rate of recurrence of IM and dysplasia after RFA ± EMR for the management of nondysplastic and dysplastic BE or early intramucosal cancer. The authors also compared the efficacy of RFA ± EMR (RFA) vs. stepwise endoscopic treatment (SRER) for the management of dysplastic BE.

The pooled incidence for any recurrence, recurrence of IM, and dysplasia was 7.5/100 patient years (PY), 4.8/100 PY, and 2.0/100 PY, respectively. The recurrence rate differed between those who underwent SRER vs. RFA ± EMR. The overall recurrence rate and the IM recurrence rate were increased after RFA ± EMR vs. stepwise treatment: 8.6/100 PY vs. 4.9/100 PY and 5.8/100 PY vs. 3.3/100 PY for stepwise vs. RFA ± EMR treatment, respectively [9].

The authors could not find a uniform typical risk profile for recurrent disease that was shared by all studies. However, factors fostering recurrence of BE after endoscopic treatment include esophagitis, presence of hiatal hernia and increased hernia size, CLE length, number of treatment sessions required to achieve IM-negative CLE, age, non-Caucasian background, smoking, decreased body mass index (25.3 vs. 29.8 for recurrence vs. no recurrence, respectively), and residual acidic reflux [9].

In their meta-analysis, Qumseya et al. [10] compared the efficacy of RFA vs. surveillance to prevent progression to HGD and cancer in patients with LGD BE. The final analysis included 19 out of 2029 cited studies. Calculations with fixed-effects models showed that RFA caused an 86% reduction in the risk of progression, when compared with surveillance. Including the data of 2746 patients, the random-effects models showed that the cumulative progression rate was 12.6% vs. 1.7% for surveillance vs. RFA, respectively. Finally, the number of RFAs required to prevent one case of HGD or cancer was 9.2; thus, fewer than ten RFAs prevents one case of HGD or cancer in patients with LGD. Therefore, the data of the study by Qumseya et al. [10] reconfirm the recommendation of the recent expert panel meeting [3].

### Management of nondysplastic BE

None of the studies explicitly examined the effect of RFA ± ER on nondysplastic BE. However, the data on nondysplastic BE included in the aforementioned studies [4–10] support following the recent recommendation [3]. Thus, RFA should be offered to persons with BE and an increased cancer risk profile, which includes GERD for more than 10 years, hiatal hernia >3.0 cm, esophagitis, BE length, and history of dysplasia. However, at present, RFA for nondysplastic BE should be exclusively performed in controlled clinical trials.

### Antireflux surgery for BE

The role of antireflux surgery and dietary aspects for the management of GERD and BE remains to be examined.

Conceptually, BE results from the impaired function or loss of function of the antireflux mechanism within the lower end of the esophagus. As a consequence, reflux occurs and fosters the development of GERD and BE [1]. Thus, it seems justified to consider the impact of treating the cause of the disease, e. g., repair of the antireflux mechanism (i. e., lower esophageal sphincter, diastasis of the musculature of the cura of the diaphragm, and formation of hiatal hernia). Medical therapy alters the acidity of the reflux, but does not alter the amount of reflux per se [11–13]. By contrast, Knight et al. [11] and Skrobic et al. [12] demonstrated that functional, effective antireflux surgery fosters the regression of BE and improves the efficacy of RFA in BE segments >4.0 cm [11, 12]. Thus, the recent data support antireflux surgery for symptomatic GERD and BE before, during, or after RFA [3, 13]. Esophageal manometry and reflux monitoring are recommended for an accurate diagnosis [2, 3, 11, 12].

Recent data showed that regular consumption of food and beverages rich in sugars and sweeteners positively correlates with obesity and GERD [14, 15]. New data published in 2017 extended this notion to BE and cancer. Li et al. found a positive correlation between a sugar-rich diet and the development of BE [16] and cancer [17]. Sugar-rich nutrition increased the incidence of BE and cancer by 70–79% [16] and 51–58% [17], respectively. Thus, nutrition seems to be of relevance in the development of GERD, BE, and cancer [14–17].

## Discussion

Here, we provide an update of the recommendations of a recent interdisciplinary expert panel meeting on the management of BE held in 2015 [3].

Conceptually, endoscopic therapies aim to prevent the progression of BE to cancer. In line with this notion, the panel recommended RFA ± EMR for BE with early cancer, HGD, and LGD [3]. This recommendation is supported by recent studies and meta-analyses published in 2016 and 2017 [4–10]. When compared with surveillance, RFA is significantly more effective in preventing the progression to cancer [10]. However, recurrence occurs in 10–25% of the cases and warrants accurate surveillance [4, 6, 7]. Major risk factors for recurrence and progression to cancer after endoscopic therapy include markers of advanced stages of the disease including increased BE length (>3 cm), baseline diagnosis of dysplasia, esophagitis, and large hiatal hernia [4, 6–10]. In accordance with the recent recommendations [3], surveillance should be timed on the basis of the baseline histopathology and should be in 3‑ and 3–6-month intervals for HGD or early cancer and LGD, respectively. Follow-up RFA is recommended for the elimination of recurrent disease [3, 4, 7, 9, 10].

There are discrepancies in the literature on how to manage nondysplastic BE [2]. Based on the recommendations of the expert panel, the published literature justifies RFA for nondysplastic BE within academic trials on patients with an increased cancer risk profile (GERD for more than 10 years, hiatal hernia >3.0 cm, esophagitis, BE length, and history of dysplasia; [3]). Recent studies focused on the treatment of dysplastic BE. However, these studies show that a higher stage of the disease (LGD, HGD) at baseline increases the probability for progression to cancer [4, 6, 7]. By contrast, RFA harbors a 90–100% chance for long-term elimination of nondysplastic BE [8]. Therefore, it seems reasonable to recommend RFA for nondysplastic BE in persons with an increased risk profile (as described earlier) in controlled academic trials [3]. After RFA, patients are kept on proton pump inhibitor (PPI) therapy [2].

Antireflux surgery targets the *cause *of the disease (impaired function of the antireflux mechanism, hiatal hernia) and stops increased exposure of the esophagus with the *mediator* of the disease (reflux), which in turn attacks the esophageal mucosa fostering the development of BE, dysplasia, and cancer [1, 11, 12]. In line with this view, a few new studies examined the effect of antireflux surgery on the treatment of BE. These studies showed that effective, functional antireflux surgery fosters regression of BE and support the efficacy of RFA [2, 11, 12]. These data are promising and warrant future studies to examine the efficacy and durability of endoscopic and surgical antireflux surgery for the management of BE and cancer prevention [3, 11]. Currently, the literature supports offering antireflux surgery to patients with GERD-symptom-positive BE before, during, or after RFA [2, 3, 11–13]. However, antireflux surgery should not be performed without an accurate diagnosis of esophageal function (manometry) and reflux monitoring [2, 3, 11–13].

Recent studies indicate the supportive role of diet and nutrition for the management of GERD [14, 16]. In 2017, this knowledge was extended to BE and esophageal cancer. In two studies, Li et al. [16, 17] demonstrated that the consumption of food and beverages containing sugars, sweeteners, and artificial sugars correlates with a 51–79% increased risk for the development of BE and adenocarcinoma of the esophagus [16, 17]. Thus, it is justified to recommended the inclusion of a low-carbohydrate diet in the management of GERD and BE.

## Conclusion

In summary, the endoscopic management (RFA ± EMR) of dysplastic BE offers an accurate pretreatment diagnosis, is approximately tenfold superior to surveillance for cancer prevention [10], warrants accurate follow-up (± RFA ± endoscopic surgery; [4, 6]), and should be conducted in expert specialized centers [3]. The efficacy of RFA for cancer prevention in nondysplastic BE should be tested in prospective academic studies. The outcome of studies investigating the impact of antireflux surgery before, during, and after RFA for cancer prevention is awaited [2, 3, 11, 12]. Finally, the recent literature supports the inclusion of a low-carbohydrate diet in the management of GERD and BE [16, 17]. Therefore, the management of BE requires a well-orchestrated multidisciplinary approach for the benefit of our patients.
